# Publisher Correction: Population genomics confirms acquisition of drug-resistant *Aspergillus fumigatus* infection by humans from the environment

**DOI:** 10.1038/s41564-022-01160-6

**Published:** 2022-06-07

**Authors:** Johanna Rhodes, Alireza Abdolrasouli, Katie Dunne, Thomas R. Sewell, Yuyi Zhang, Eloise Ballard, Amelie P. Brackin, Norman van Rhijn, Harry Chown, Alexandra Tsitsopoulou, Raquel B. Posso, Sanjay H. Chotirmall, Noel G. McElvaney, Philip G. Murphy, Alida Fe Talento, Julie Renwick, Paul S. Dyer, Adrien Szekely, Paul Bowyer, Michael J. Bromley, Elizabeth M. Johnson, P. Lewis White, Adilia Warris, Richard C. Barton, Silke Schelenz, Thomas R. Rogers, Darius Armstrong-James, Matthew C. Fisher

**Affiliations:** 1 Medical Research Council Centre for Global Disease Analysis, Imperial College London, London, UK; 2 Department of Infectious Diseases, Imperial College London, London, UK; 3 Department of Medical Microbiology, King’s College University Hospital, London, UK; 4 Department of Clinical Microbiology, Trinity College Dublin, Dublin, Ireland; 5 Aberdeen Fungal Group, Institute of Medical Sciences, University of Aberdeen, Aberdeen, UK; 6 Manchester Fungal Infection Group, Faculty of Biology, Medicine and Health, The University of Manchester, Manchester Academic Health Science Centre, Core Technology Facility, Manchester, UK; 7 Microbiology Department, Royal Glamorgan Hospital, Cwm Taf NHS Trust, Ynysmaerdy, UK; 8 Public Health Wales Microbiology, Cardiff, UK; 9 Lee Kong Chian School of Medicine, Nanyang Technological University, Singapore, Singapore; 10 Respiratory Research Division, Department of Medicine, Royal College of Surgeons in Ireland, Education and Research Centre, Beaumont Hospital, Dublin, Ireland; 11 Department of Medical Microbiology, Tallaght University Hospital, Dublin, Ireland; 12 School of Life Sciences, University of Nottingham, Nottingham, UK; 13 Head Mycology Reference Laboratory, UK Health Security Agency, Bristol, UK; 14 Medical Research Council Centre for Medical Mycology, University of Exeter, Exeter, UK; 15 Mycology Reference Centre, Leeds Teaching Hospitals National Health Service Trust, Leeds, UK; 16 Infection Sciences, Kings College University Hospital, London, UK

**Keywords:** Genome evolution, Rare variants

Correction to: *Nature Microbiology*10.1038/s41564-022-01091-2, published online 25 April 2022.

In the version of this article initially published, a production error resulted in partially duplicated circles representing the various isolates in the phylogenetic analysis of Fig. 1c.The figure has been corrected in the HTML and PDF versions of the article.

