# Supplementary material for: Population genomics confirms acquisition of drug-resistant Aspergillus fumigatus infection by humans from the environment
Source: Nat Microbiol. 2022 Apr 25;7(5):663–74. doi: 10.1038/s41564-022-01091-2 (PMC9064804; doi:10.1038/s41564-022-01091-2)
Supplement: Supplementary file 1 — Supplementary Tables 1–10 and Data 1–7. [file 41564_2022_1091_MOESM1_ESM.pdf]

nature portfolio

https://doi.org/10.1038/s41564-022-01091-2

Supplementary information

Population genomics confirms acquisition of drug-resistant *Aspergillus fumigatus* infection by humans from the environment

In the format provided by the authors and unedited

Supplementary Information

Supplementary Tables

Table S1

Clinical and environmental isolates of *A. fumigatus* used in this study, and details of whole-genome alignments. Patient cohort details for clinical isolates is provided, when available. Location information provided, where available. More accurate latitude and longitude details provided in the Microreact project. CF = cystic fibrosis; COPD = chronic obstructive pulmonary disease; RLL = right lower lobe; ABPA = allergic bronchopulmonary aspergillosis.

Isolate	Source	Location	*Cyp51A* polymorphisms	No. of reads aligned (millions)	Depth of coverage (*x*)	% reference genome covered
ARAF001	Clinical (CF)	Leeds, England	TR_{34}/L98H	51.9	174	94.4
ARAF002	Clinical (CF)	Leeds, England	TR_{34}/L98H	48.3	162	95.0
ARAF003	Clinical (CF)	Leeds, England	Wildtype	43.6	147	95.2
ARAF004	Clinical (CF)	Leeds, England	Wildtype	43.7	146	93.1
ARAF005	Clinical (CF)	Leeds, England	Wildtype	47.4	159	95.4
ARAF006	Clinical (CF)	Leeds, England	Wildtype	50.1	169	93.2
C1	Environmental	Cardiff, Wales	TR_{34}/L98H	6.1	31	91.1
C2	Environmental	Cardiff, Wales	TR_{34}/L98H	7.2	36	89.6
C3	Environmental	Cardiff, Wales	TR_{34}/L98H	6.5	33	91.1
C4	Environmental	Cardiff, Wales	Wildtype	7.5	37	91.9
C5	Environmental	Vale of Glamorgan, Wales	TR_{34}/L98H	6.7	33	91.1
C6	Environmental	Vale of Glamorgan, Wales	TR_{34}/L98H	7.3	37	89.2
C7	Environmental	Vale of Glamorgan, Wales	TR_{34}/L98H	6.4	32	88.6
C8	Environmental	Vale of Glamorgan, Wales	TR_{34}/L98H	6.8	34	91.2
C10	Environmental	Carmerthenshire, Wales	TR_{34}/L98H	7.0	35	88.9
C11	Environmental	Carmerthenshire, Wales	TR_{34}/L98H	7.4	37	89.1
C12	Environmental	Carmerthenshire, Wales	TR_{34}/L98H	6.0	34	91.1

C13	Environmental	Vale of Glamorgan, Wales	TR_{34}/L98H	7.1	36	90.9
C14	Environmental	Vale of Glamorgan, Wales	TR_{34}/L98H	7.6	38	90.8
C15	Environmental	Vale of Glamorgan, Wales	TR_{34}/L98H	7.4	37	90.7
C16	Environmental	Vale of Glamorgan, Wales	TR_{34}/L98H	6.8	34	90.8
C17	Environmental	Vale of Glamorgan, Wales	TR_{34}/L98H	7.5	37	91.2
C18	Environmental	Vale of Glamorgan, Wales	TR_{34}/L98H	6.4	32	90.6
C19	Environmental	Vale of Glamorgan, Wales	TR_{34}/L98H	6.5	32	89.0
C20	Environmental	Vale of Glamorgan,	TR_{34}/L98H	7.0	35	91.1
C21	Environmental	Pembrokeshire, Wales	TR_{34}/L98H	6.9	34	91.1
C22	Environmental	Newport, Wales	TR_{34}/L98H	7.4	37	91.1
C23	Environmental	Newport, Wales	TR_{34}/L98H	7.4	37	91.2
C24	Environmental	Newport, Wales	TR_{34}/L98H	6.1	31	90.5
C25	Environmental	Cardiff, Wales	TR_{34}/L98H	7.0	35	87.7
C26	Environmental	Cardiff, Wales	G54R	5.3	26	87.9
C27	Environmental	Cardiff, Wales	TR_{34}/L98H	6.2	31	88.3
C28	Environmental	Cardiff, Wales	TR_{34}/L98H	5.8	29	90.7
C29	Environmental	Cardiff, Wales	TR_{34}/L98H	5.8	29	90.9
C30	Environmental	Cardiff, Wales	TR_{34}/L98H	5.9	29	90.7
C31	Environmental	Monmouthshire, Wales	TR_{34}/L98H	6.4	32	90.8
C32	Clinical (CF)	Cardiff, Wales	TR_{34}/L98H	6.6	33	91.1
C33	Clinical (CF)	Cardiff, Wales	TR_{34}/L98H	6.1	30	91.1
C34	Clinical (CF)	Cardiff, Wales	Wildtype	6.5	32	90.9
C35	Clinical (CF)	Cardiff, Wales	Wildtype	6.6	33	88.8
C36	Clinical (CF)	Cardiff, Wales	Wildtype	6.6	33	88.9
C37	Clinical (CF)	Cardiff, Wales	Wildtype	7.1	36	90.7

C38	Clinical (CF)	Cardiff, Wales	Wildtype	7.1	35	89.3
C39	Clinical (CF)	Cardiff, Wales	Wildtype	6.9	35	88.6
C40	Clinical (CF)	Dublin, Rep. of Ireland	Wildtype	7.3	37	89.3
C41	Clinical (CF)	Dublin, Rep. of Ireland	Wildtype	7.7	39	89.4
C42	Clinical (CF)	Dublin, Rep. of Ireland	Wildtype	6.7	33	91.8
C43	Clinical (CF)	Dublin, Rep. of Ireland	Wildtype	6.6	33	91.8
C44	Clinical (CF)	Dublin, Rep. of Ireland	Wildtype	6.1	31	91.7
C45	Clinical (CF)	Dublin, Rep. of Ireland	Wildtype	6.0	30	93.4
C46	Clinical (CF)	Dublin, Rep. of Ireland	Wildtype	6.5	33	91.2
C47	Clinical (CF)	Dublin, Rep. of Ireland	Wildtype	7.1	35	92.2
C48	Clinical (CF)	Dublin, of Ireland	Wildtype	7.1	36	91.8
C49	Clinical (CF)	Dublin, Rep. of Ireland	Wildtype	7.6	38	88.9
C50	Clinical (CF)	Dublin, Rep. of Ireland	Wildtype	8.4	42	88.8
C51	Clinical (CF)	Dublin, Rep. of Ireland	Wildtype	0.5	37	93.3
C52	Clinical (CF)	Dublin, Rep. of Ireland	Wildtype	7.5	37	89.9
C53	Clinical (CF)	Dublin, Rep. of Ireland	Wildtype	7.0	35	91.3
C54	Clinical (CF)	Dublin, Rep. of Ireland	Wildtype	6.6	33	92.4
C55	Clinical (CF)	Dublin, Rep. of Ireland	Wildtype	8.0	40	91.6
C56	Clinical (CF)	Dublin, Rep. of Ireland	Wildtype	7.3	36	91.3
C57	Clinical (CF)	Dublin, Rep. of Ireland	Wildtype	7.6	38	91.4
C58	Clinical (CF)	Dublin, Rep. of Ireland	Wildtype	6.1	31	90.6
C59	Clinical (CF)	Dublin, Rep. of Ireland	Wildtype	7.3	37	90.1
C60	Clinical (CF)	Dublin, Rep. of Ireland	Wildtype	6.5	33	90.1
C61	Clinical (CF)	Dublin, Rep. of Ireland	Wildtype	6.9	35	90.4
C62	Clinical (CF)	Dublin, Rep. of Ireland	Wildtype	7.0	35	90.1

C63	Clinical (CF)	Dublin, Rep. of Ireland	Wildtype	7.1	35	90.1
C64	Clinical (CF)	Dublin, Rep. of Ireland	Wildtype	7.1	35	89.8
C65	Clinical (CF)	Dublin, Rep. of Ireland	Wildtype	7.4	37	89.6
C66	Clinical (CF)	Dublin, Rep. of Ireland	Wildtype	6.6	33	89.5
C67	Clinical	Dublin, Rep. of Ireland	Wildtype	6.2	31	91.1
C68	Clinical (CF)	Dublin, Rep. of Ireland	TR_{34}/L98H	6.3	32	91.1
C69	Clinical	Dublin, Rep. of Ireland	TR_{34}/L98H	6.9	35	91.2
C70	Clinical	Dublin, Rep. of Ireland	TR_{34}/L98H	6.4	32	91.2
C71	Clinical	Dublin, Rep. of Ireland	Wildtype	6.5	32	90.0
C72	Clinical	Dublin, Rep. of Ireland	Wildtype	7.4	37	90.0
C73	Clinical	Dublin, Rep. of Ireland	Wildtype	7.3	37	90.4
C74	Clinical	Dublin, Rep. of Ireland	Wildtype	7.7	38	89.3
C75	Clinical	Dublin, Rep. of Ireland	Wildtype	8.5	42	89.7
C76	Clinical	Dublin, Rep. of Ireland	Wildtype	7.4	37	88.9
C77	Clinical	Dublin, Rep. of Ireland	Wildtype	7.5	37	89.0
C78	Environmental	Dublin, Rep. of Ireland	TR_{34}/L98H	8.1	40	89.1
C79	Environmental	Dublin, Rep. of Ireland	TR_{46}/T289A/Y121F	8.1	40	91.9
C80	Environmental	Dublin, Rep. of Ireland	TR_{34}/L98H	7.8	39	89.5
C81	Clinical	Dublin, Rep. of Ireland	Wildtype	8.7	43	91.7
C82	Clinical	Dublin, Rep. of Ireland	TR_{34}/L98H	6.6	33	88.2
C83	Clinical	Dublin, Rep. of Ireland	TR_{34}/L98H	8.2	41	91.3
C84	Clinical	Dublin, Rep. of Ireland	TR_{34}/L98H	6.8	34	91.2
C85	Environmental	Dublin, Rep. of Ireland	TR_{34}/L98H	7.1	35	90.9
C86	Environmental	Dublin, Rep. of Ireland	TR_{34}/L98H	7.2	36	92.0
C87	Environmental	Dublin, Rep. of Ireland	TR_{46}/T289A/Y121F	8.0	40	90.1

C88	Environmental	Dublin, Rep. of Ireland	TR_{34}/L98H	7.4	37	93.3
C89	Environmental	Dublin, Rep. of Ireland	TR_{46}/T289A/Y121F	7.2	36	90.4
C91	Environmental	Dublin, Rep. of Ireland	TR_{46}/T289A/Y121F	6.8	34	90.1
C92	Environmental	Dublin, Rep. of Ireland	TR_{34}/L98H	7.3	37	90.8
C93	Environmental	Dublin, Rep. of Ireland	TR_{46}/T289A/Y121F	6.6	33	90.7
C95	Environmental	Dublin, Rep. of Ireland	Wildtype	7.3	36	89.7
C96	Environmental	Dublin, Rep. of Ireland	Wildtype	7.6	38	91.9
C99	Clinical	Scotland	TR_{34}/L98H	5.8	28	96.0
C100	Clinical (CF)	England	TR_{34}/L98H	6.3	31	96.2
C103	Clinical	England	TR_{34}/L98H	5.8	28	96.5
C104	Clinical	Scotland	TR_{34}/L98H	5.7	28	95.9
C105	Clinical	Scotland	TR_{34}/L98H	5.6	27	95.6
C106	Clinical	England	TR_{34}/L98H	6.0	29	96.5
C107	Clinical	England	TR_{34}/L98H	6.5	32	95.7
C108	Clinical	England	TR_{34}/L98H	6.8	33	96.6
C109	Clinical	England	TR_{34}/L98H	6.1	29	96.5
C110	Clinical (CF)	England	TR_{34}/L98H	6.3	31	95.7
C111	Clinical (COPD)	England	TR_{34}/L98H	6.6	32	96.6
C112	Clinical (CF)	England	TR_{34}/L98H	6.5	32	96.2
C113	Clinical	England	Wildtype	6.2	30	96.1
C114	Clinical (Bronchiectasis)	Scotland	TR_{34}/L98H	6.6	32	96.0
C115	Clinical (Liver failure)	England	TR_{34}/L98H	6.5	30	96.5
C117	Clinical	England	Wildtype	6.2	30	94.6
C118	Clinical	England	Wildtype	6.6	31	95.1

C119	Clinical (respiratory illness/TB)	England	TR_{34}/L98H	6.2	30	95.6
C120	Clinical (Lung lesion)	England	G54R	6.4	31	96.6
C121	Clinical	England	G54E	6.1	31	94.9
C122	Clinical (RLL cavity)	England	TR_{34}/L98H	6.3	31	95.7
C123	Clinical	England	Wildtype	6.2	29	96.2
C124	Clinical	London, England	G54W	6.3	29	96.8
C125	Clinical (CF)	London, England	Wildtype	6.3	27	96.2
C126	Clinical (Aortic valve replacement)	London, England	G54W	6.5	28	96.8
C127	Clinical (CF)	London, England	Wildtype	6.0	29	96.6
C128	Clinical (CF)	London, England	Wildtype	5.5	27	97.1
C129	Clinical (CF)	London, England	Wildtype	5.6	27	96.1
C130	Clinical (CF)	London, England	G54W	5.8	28	96.4
C131	Clinical (CF)	London, England	G54W	6.0	29	96.4
C132	Clinical (CF)	London, England	G54W	6.5	32	96.4
C133	Clinical	London, England	TR_{34}/L98H	5.9	29	96.2
C134	Clinical (CF)	London, England	TR_{34}/L98H	6.1	30	96.7
C135	Clinical (CF)	London, England	G54W	6.4	31	96.4
C136	Clinical (CF)	London, England	Wildtype	5.8	28	97.0
C137	Clinical (CF)	London, England	Wildtype	5.9	29	97.1
C138	Clinical (ABPA)	London, England	P216L	6.3	31	96.4
C139	Clinical (CF)	London, England	Wildtype	6.3	31	96.2
C140	Clinical (Bronchiectasis)	London, England	G54W	6.5	32	96.8
C141	Clinical (CF)	London, England	TR_{34}/L98H	6.4	31	96.8

C142	Clinical (Bronchiectasis)	London,	G54W	6.5	31	99.6
C143	Clinical (CF)	London, England	TR_{34}/L98H	6.4	31	96.2
C144	Clinical (CF)	London,	TR_{34}/L98H	6.3	30	95.8
C145	Clinical (CF)	London, England	P216L	6.2	30	96.9
C146	Clinical (CF)	Leeds, England	Wildtype	6.9	34	96.9
C147	Clinical (CF)	Leeds, England	Wildtype	7.2	35	96.9
C148	Clinical (CF)	Leeds, England	Wildtype	7.3	35	96.7
C149	Clinical (CF)	Leeds, England	TR_{34}/L98H	6.9	34	96.7
C150	Clinical (CF)	Leeds, England	TR_{34}/L98H	7.2	35	96.7
C151	Clinical (CF)	Leeds,	TR_{34}/L98H	6.8	33	96.7
C152	Clinical (CF)	Leeds, England	Wildtype	7.0	34	96.9
C153	Clinical (CF)	Leeds, England	Wildtype	6.9	34	96.9
C154	Clinical (CF)	Leeds, England	Wildtype	7.0	34	96.9
C155	Clinical	London, England	TR_{34}/L98H/T289A/I364V/G448S	7.0	34	95.4
C156	Clinical	London, England	TR_{34}/L98H/T289A/I364V/G448S	6.4	31	95.4
C157	Clinical	London, England	TR_{34}/L98H/T289A/I364V/G448S	6.3	31	95.4
C158	Clinical	London, England	TR_{34}/L98H/T289A/I364V/G448S	6.2	31	95.4
C159	Clinical (Asthma and bronchiectasis)	London, England	TR_{34}/L98H	6.3	31	95.7
C160	Clinical (Asthma and bronchiectasis)	London, England	TR_{34}/L98H	6.3	31	95.7
C161	Clinical (trauma)	London, England	TR_{34}/L98H	6.3	31	96.5
C162	Clinical	London, England	Wildtype	6.6	32	94.9
C163	Clinical	London, England	G54W	6.6	32	95.0
C164	Clinical (CF)	London, England	Wildtype	6.4	31	96.6

C165	Clinical (CF)	London, England	Wildtype	6.4	32	95.4
C166	Clinical (CF)	London, England	Wildtype	6.6	32	96.2
C167	Clinical (CF)	London, England	Wildtype	6.5	32	96.5
C168	Clinical (CF)	London, England	Wildtype	6.5	32	96.0
C169	Clinical (CF)	London, England	Wildtype	6.4	31	96.2
C170	Clinical (CF)	London, England	Wildtype	6.4	32	96.3
C171	Clinical (CF)	London, England	TR_{34}/L98H	7.3	36	96.9
C172	Clinical (CF)	London, England	Wildtype	7.4	34	96.0
C173	Clinical (CF)	London, England	Wildtype	7.3	36	95.1
C174	Clinical (CF)	London, England	Wildtype	7.7	38	95.1
C175	Clinical (CF)	London, England	Wildtype	7.1	35	97.0
C176	Clinical (CF)	London, England	Wildtype	7.0	34	97.0
C177	Clinical (CF)	London, England	Wildtype	7.6	37	94.9
C178	Clinical (CF)	London, England	Wildtype	7.2	35	97.9
C179	Clinical (CF)	London, England	Wildtype	7.5	37	95.9
C180	Clinical (CF)	London, England	Wildtype	5.8	28	97.0
C181	Clinical (CF)	London, England	Wildtype	6.6	32	96.1
C182	Clinical (CF)	London, England	Wildtype	7.2	35	96.2
C183	Clinical (CF)	London, England	Wildtype	6.8	33	96.3
C184	Clinical (CF)	London, England	Wildtype	6.9	33	96.9
C185	Clinical (CF)	London, England	Wildtype	6.8	33	96.9
C186	Clinical (CF)	London, England	Wildtype	7.0	34	96.6
C187	Clinical (CF)	London, England	Wildtype	6.7	33	96.2
C188	Clinical (CF)	London, England	Wildtype	7.1	35	94.8
C189	Clinical (CF)	London, England	G54R	6.6	32	96.9

C190	Clinical (CF)	London, England	Wildtype	6.5	32	96.5
C191	Clinical (CF)	London, England	Wildtype	6.8	33	97.2
C220	Environmental	Dublin, Rep. of Ireland	Wildtype	6.6	30	95.1
C221	Environmental	Dublin, Rep. of Ireland	Wildtype	7.0	31	94.7
C222	Environmental	Dublin, Rep. of Ireland	Wildtype	10.3	37	95.2
C223	Environmental	Dublin, Rep. of Ireland	Wildtype	7.8	35	94.9
C246	Environmental	Nottingham, England	Wildtype	9.7	42	94.7
C272	Environmental	Nottingham, England	Wildtype	9.4	42	95.9
C275	Environmental	Yorkshire, England	Wildtype	8.1	36	96.8
C341	Environmental	Didcot, England	TR_{34}/L98H	8.5	42	96.7
C342	Environmental	Hyde Park, England	TR_{34}/L98H	9.9	48	95.4
C343	Environmental	Hyde Park, England	Wildtype	9.0	44	96.3
C344	Environmental	Hyde Park, England	Wildtype	9.1	45	96.0
C345	Environmental	Hyde Park, England	Wildtype	8.9	44	96.6
C346	Environmental	Hyde Park, England	Wildtype	8.6	42	96.1
C354	Clinical	Dublin, Rep. of Ireland	TR_{34}/L98H	9.1	45	96.0
C355	Clinical	Dublin, Rep. of Ireland	TR_{34}/L98H	9.9	48	96.0
C356	Clinical	Dublin, Rep. of Ireland	TR_{34}/L98H	8.9	44	96.7
C357	Environmental	Dublin, Rep. of Ireland	TR_{34}/L98H	8.9	44	96.0
C358	Environmental	Dublin, of Ireland	TR_{46}/T289A/Y121F	9.3	45	96.3
C359	Clinical	Dublin, Rep. of Ireland	TR_{34}/L98H	10.3	50	97.0
C360	Environmental	Dublin, Rep. of Ireland	TR_{46}/T289A/Y121F	8.7	42	96.2
C361	Clinical	Dublin, Rep. of Ireland	Wildtype	9.0	44	96.4
C362	Clinical	Dublin, Rep. of Ireland	Wildtype	8.4	41	96.4
C363	Clinical	Dublin, Rep. of Ireland	Wildtype	9.2	45	96.5

C364	Environmental	Aberdeen, Scotland	TR_{34}/L98H	8.8	43	96.9
C365	Environmental	Aberdeen, Scotland	TR_{34}	10.0	49	96.8
C366	Environmental	Aberdeen, Scotland	TR_{34}/L98H	10.3	49	96.3
C367	Environmental	Aberdeen, Scotland	Wildtype	10.2	50	95.8
C368	Environmental	Aberdeen, Scotland	TR_{34}/L98H	10.4	48	96.3
C369	Environmental	Aberdeen, Scotland	TR_{34}/L98H	9.2	45	96.3

Table S2

In vitro antifungal susceptibility profiles of *A. fumigatus* isolates and corresponding resistance markers in *cyp51A*, mating type idiomorph and Clade membership as detected by whole genome sequencing. ND = not determined.

Isolate	MIC (mg/litre) of[a]:			Resistance marker	Mating type	Clade membership
	ITC	VOR	POS			
ARAF001	>16	1	0.5	TR_{34}/L98H	MAT1-1	A
ARAF002	>16	1	0.5	TR_{34}/L98H	MAT1-1	A
ARAF003	1	0.25	0.06	Wildtype	MAT1-2	B
ARAF004	1	0.5	0.25	Wildtype	MAT1-1	A
ARAF005	0.5	0.125	0.06	Wildtype	MAT1-2	B
ARAF006	0.5	0.5	0.06	Wildtype	MAT1-1	A
C1	>16	2	0.12	TR_{34}/L98H	MAT1-2	A
C2	>16	2	0.25	TR_{34}/L98H	MAT1-2	A
C3	>16	2	0.12	TR_{34}/L98H	MAT1-2	A
C4	>16	8	<0.5	Wildtype	MAT1-2	B
C5	>16	2	0.12	TR_{34}/L98H	MAT1-2	A
C6	>16	2	0.25	TR_{34}/L98H	MAT1-2	A
C7	>16	4	0.25	TR_{34}/L98H	MAT1-2	A
C8	>16	2	0.12	TR_{34}/L98H	MAT1-2	A
C10	>16	8	0.5	TR_{34}/L98H	MAT1-2	A
C11	>16	2	0.25	TR_{34}/L98H	MAT1-2	A
C12	>16	4	0.25	TR_{34}/L98H	MAT1-2	A
C13	>16	2	0.12	TR_{34}/L98H	MAT1-2	A
C14	>16	>8	0.5	TR_{34}/L98H	MAT1-2	A
C15	>16	8	<0.5	TR_{34}/L98H	MAT1-2	A
C16	>16	2	0.12	TR_{34}/L98H	MAT1-2	A
C17	>16	2	0.12	TR_{34}/L98H	MAT1-2	A
C18	>16	2	0.12	TR_{34}/L98H	MAT1-2	A
C19	>16	1	0.25	TR_{34}/L98H	MAT1-2	A
C20	>16	2	<0.25	TR_{34}/L98H	MAT1-2	A
C21	>16	2	0.025	TR_{34}/L98H	MAT1-2	A

C22	>16	2	0.12	TR_{34}/L98H	MAT1-2	A
C23	>16	2	0.12	TR_{34}/L98H	MAT1-2	A
C24	>16	2	0.12	TR_{34}/L98H	MAT1-2	A
C25	>16	4	0.25	TR_{34}/L98H	MAT1-2	A
C26	>16	0.25	1	G54R	MAT1-2	A
C27	>16	1	0.25	TR_{34}/L98H	MAT1-2	A
C28	>16	4	0.25	TR_{34}/L98H	MAT1-1	A
C29	>16	4	0.5	TR_{34}/L98H	MAT1-2	A
C30	>16	2	0.25	TR_{34}/L98H	MAT1-2	A
C31	>16	2	0.12	TR_{34}/L98H	MAT1-2	A
C32	>16	0.5	0.5	TR_{34}/L98H	MAT1-2	A
C33	>16	1	0.5	TR_{34}/L98H	MAT1-2	A
C34	<0.03	0.06	0.03	Wildtype	MAT1-1	B
C35	0.06	0.06	0.06	Wildtype	MAT1-1	A
C36	0.06	0.25	0.06	Wildtype	MAT1-1	A
C37	0.25	0.125	0.06	Wildtype	MAT1-1	B
C38	0.125	0.25	0.06	Wildtype	MAT1-1	A
C39	0.06	0.25	0.06	Wildtype	MAT1-2	B
C40	0.06	0.25	0.03	Wildtype	MAT1-1	B
C41	0.25	0.25	0.25	Wildtype	MAT1-1	B
C42	0.25	0.25	0.12	Wildtype	MAT1-2	B
C43	0.03	0.12	0.015	Wildtype	MAT1-2	B
C44	0.03	0.12	0.015	Wildtype	MAT1-2	B
C45	0.03	0.12	0.015	Wildtype	MAT1-2	B
C46	0.03	0.12	0.015	Wildtype	MAT1-2	B
C47	0.03	0.015	0.015	Wildtype	MAT1-2	B
C48	0.03	0.015	0.015	Wildtype	MAT1-2	B
C49	0.12	0.25	0.06	Wildtype	MAT1-1	B
C50	0.25	0.12	0.06	Wildtype	MAT1-1	B
C51	0.06	0.25	0.03	Wildtype	MAT1-2	B
C52	<0.015	0.12	0.03	Wildtype	MAT1-2	B
C53	0.25	0.12	0.06	Wildtype	MAT1-2	B
C54	0.06	0.25	0.03	Wildtype	MAT1-2	B
C55	0.03	0.25	0.015	Wildtype	MAT1-2	B
C56	0.12	0.12	0.015	Wildtype	MAT1-1	B
C57	0.06	0.06	0.03	Wildtype	MAT1-1	B
C58	0.03	0.12	0.015	Wildtype	MAT1-1	A
C59	0.25	0.12	0.06	Wildtype	MAT1-1	A
C60	0.12	0.12	0.03	Wildtype	MAT1-1	A
C61	0.03	0.25	0.015	Wildtype	MAT1-1	A
C62	0.03	0.25	0.015	Wildtype	MAT1-1	A
C63	0.03	0.12	0.015	Wildtype	MAT1-1	A
C64	0.25	0.25	0.12	Wildtype	MAT1-1	A
C65	0.03	0.12	0.015	Wildtype	MAT1-1	A
C66	0.03	0.12	0.015	Wildtype	MAT1-1	A
C67	0.03	0.25	0.015	Wildtype	MAT1-2	B
C68	0.25	0.25	0.06	TR_{34}/L98H	MAT1-2	A
C69	1	0.5	0.25	TR_{34}/L98H	MAT1-2	A
C70	1	1	0.25	TR_{34}/L98H	MAT1-2	A
C71	0.12	0.03	0.008	Wildtype	MAT1-1	B

C72	0.03	0.12	0.015	Wildtype	MAT1-1	B
C73	0.03	0.12	0.015	Wildtype	MAT1-2	B
C74	0.03	0.12	0.015	Wildtype	MAT1-2	B
C75	0.25	0.25	0.12	Wildtype	MAT1-1	A
C76	0.03	0.12	0.015	Wildtype	MAT1-2	B
C77	0.06	0.25	0.03	Wildtype	MAT1-2	B
C78	ND	ND	ND	TR$_{34}$/L98H	MAT1-1	A
C79	ND	ND	ND	TR$_{46}$/Y121F/T289A	MAT1-2	A
C80	ND	ND	ND	TR$_{34}$/L98H	MAT1-2	A
C81	0.25	0.25	0.06	Wildtype	MAT1-1	B
C82	4	2	1	TR$_{34}$/L98H	MAT1-2	A
C83	16	2	0.5	TR$_{34}$/L98H	MAT1-2	A
C84	4	4	0.5	TR$_{34}$/L98H	MAT1-2	A
C85	ND	ND	ND	TR$_{34}$/L98H	MAT1-2	A
C86	ND	ND	ND	TR$_{34}$/L98H	MAT1-1	A
C87	ND	ND	ND	TR$_{46}$/Y121F/T289A	MAT1-1	B
C88	ND	ND	ND	TR$_{34}$/L98H	MAT1-2	A
C89	ND	ND	ND	TR$_{46}$/Y121F/T289A	MAT1-2	A
C91	ND	ND	ND	TR$_{46}$/Y121F/T289A	MAT1-1	B
C92	ND	ND	ND	TR$_{34}$/L98H	MAT1-2	A
C93	ND	ND	ND	TR$_{46}$/Y121F/T289A	MAT1-2	A
C95	ND	ND	ND	Wildtype	MAT1-1	B
C96	ND	ND	ND	Wildtype	MAT1-2	B
C99	2	2	0.5	TR$_{34}$/L98H	MAT1-1	A
C100	16	4	ND	TR$_{34}$/L98H	MAT1-2	A
C103	1	2	ND	TR$_{34}$/L98H	MAT1-2	A
C104	>16	0.5	1	TR$_{34}$/L98H	MAT1-1	A
C105	>16	4	ND	TR$_{34}$/L98H	MAT1-2	A
C106	2	4	ND	TR$_{34}$/L98H	MAT1-2	A
C107	1	4	ND	TR$_{34}$/L98H	MAT1-2	A
C108	16	1	ND	TR$_{34}$/L98H	MAT1-2	A
C109	>16	1	ND	TR$_{34}$/L98H	MAT1-1	B
C110	>16	1	ND	TR$_{34}$/L98H	MAT1-2	A
C111	16	2	ND	TR$_{34}$/L98H	MAT1-1	A
C112	16	2	ND	TR$_{34}$/L98H	MAT1-2	A
C113	ND	0.25	ND	Wildtype	MAT1-1	B
C114	>16	2	ND	TR$_{34}$/L98H	MAT1-1	A
C115	4	1	0.25	TR$_{34}$/L98H	MAT1-1	A
C117	1	2	ND	Wildtype	MAT1-2	B
C118	8	1	ND	Wildtype	MAT1-2	B
C119	16	1	ND	TR$_{34}$/L98H	MAT1-2	A
C120	>16	0.125	ND	G54R	MAT1-1	B
C121	>16	0.25	0.5	G54E	MAT1-1	A
C122	>16	2	ND	TR$_{34}$/L98H	MAT1-2	A
C123	>16	2	0.25	TR$_{34}$/L98H	MAT1-2	A
C124	>16	0.125	4	G54W	MAT1-1	B
C125	>16	2	2	TR$_{34}$/L98H	MAT1-2	A
C126	>16	0.03	2	G54W	MAT1-1	B
C127	>16	0.25	0.125	Wildtype	MAT1-2	B
C128	>16	2	1	Wildtype	MAT1-2	B

C129	8	2	0.5	Wildtype	MAT1-2	B
C130	>16	0.125	8	G54W	MAT1-2	B
C131	>16	0.125	16	G54W	MAT1-2	B
C132	>16	0.125	8	G54W	MAT1-2	B
C133	>16	2	2	TR_{34}/L98H	MAT1-2	A
C134	16	2	1	TR_{34}/L98H	MAT1-1	B
C135	>16	0.125	16	G54W	MAT1-2	B
C136	>16	2	2	Wildtype	MAT1-2	B
C137	>16	2	2	Wildtype	MAT1-2	B
C138	>16	0.25	2	P216L	MAT1-1	B
C139	>16	2	1	Wildtype	MAT1-2	B
C140	>16	0.125	16	G54W	MAT1-1	B
C141	16	1	0.25	TR_{34}/L98H	MAT1-1	B
C142	>16	0.06	0.25	G54W	MAT1-1	A
C143	16	2	0.5	TR_{34}/L98H	MAT1-2	A
C144	>16	2	0.25	TR_{34}/L98H	MAT1-2	A
C145	>16	0.125	0.5	P216L	MAT1-2	B
C146	0.5	0.125	0.03	Wildtype	MAT1-2	B
C147	1	0.125	0.03	Wildtype	MAT1-2	B
C148	2	0.125	0.03	Wildtype	MAT1-2	B
C149	>16	0.25	0.125	TR_{34}/L98H	MAT1-1	A
C150	16	0.25	0.125	TR_{34}/L98H	MAT1-1	A
C151	>16	0.25	0.125	TR_{34}/L98H	MAT1-1	A
C152	0.5	0.125	0.03	Wildtype	MAT1-2	B
C153	1	0.125	0.03	Wildtype	MAT1-2	B
C154	1	0.125	0.03	Wildtype	MAT1-2	B
C155	16	>16	4	TR_{34}/L98H/T289A/I364V/G448S	MAT1-2	A
C156	16	>16	4	TR_{34}/L98H/T289A/I364V/G448S	MAT1-2	A
C157	16	>16	4	TR_{34}/L98H/T289A/I364V/G448S	MAT1-2	A
C158	16	>16	4	TR_{34}/L98H/T289A/I364V/G448S	MAT1-2	A
C159	>16	2	0.5	TR_{34}/L98H	MAT1-2	A
C160	>16	2	0.5	TR_{34}/L98H	MAT1-2	A
C161	4	0.5	0.125	TR_{34}/L98H	MAT1-1	A
C162	2	2	1	Wildtype	MAT1-1	A
C163	>16	1	>16	G54W	MAT1-1	A
C164	2	1	0.125	Wildtype	MAT1-2	B
C165	2	0.5	0.125	Wildtype	MAT1-1	B
C166	1	0.25	0.06	Wildtype	MAT1-2	B
C167	0.06	0.06	0.03	Wildtype	MAT1-1	B
C168	2	0.25	0.03	Wildtype	MAT1-1	B
C169	0.06	0.06	0.015	Wildtype	MAT1-2	B
C170	0.06	0.06	0.015	Wildtype	MAT1-2	B
C171	16	0.5	0.25	TR_{34}/L98H	MAT1-2	A
C172	0.06	0.06	0.03	Wildtype	MAT1-2	B
C173	0.06	0.06	0.015	Wildtype	MAT1-1	B
C174	0.06	0.06	0.03	Wildtype	MAT1-1	B
C175	0.06	0.06	0.03	Wildtype	MAT1-2	B
C176	0.06	0.06	0.03	Wildtype	MAT1-1	B
C177	0.06	0.125	0.03	Wildtype	MAT1-1	A
C178	0.06	0.06	0.03	Wildtype	MAT1-2	B

C179	0.06	0.06	0.03	Wildtype	MAT1-1	B
C180	0.06	0.06	0.03	Wildtype	MAT1-2	B
C181	0.06	0.125	0.06	Wildtype	MAT1-1	B
C182	0.125	0.125	0.03	Wildtype	MAT1-2	B
C183	0.125	0.06	0.03	Wildtype	MAT1-2	B
C184	0.06	0.06	0.03	Wildtype	MAT1-2	B
C185	0.06	0.06	0.03	Wildtype	MAT1-1	B
C186	0.06	0.06	0.03	Wildtype	MAT1-1	A
C187	0.06	0.06	0.03	Wildtype	MAT1-2	B
C188	0.125	0.06	0.03	Wildtype	MAT1-1	A
C189	>16	0.06	0.25	G54R	MAT1-2	B
C190	0.06	0.03	0.03	Wildtype	MAT1-1	B
C191	0.06	0.06	0.015	Wildtype	MAT1-2	B
C220	0.06	0.06	0.06	Wildtype	MAT1-1	A
C221	0.125	0.06	0.06	Wildtype	MAT1-2	A
C222	0.06	0.06	0.03	Wildtype	MAT1-2	B
C223	0.125	0.06	0.06	Wildtype	MAT1-1	A
C246	0.125	0.125	0.06	Wildtype	MAT1-2	A
C272	0.125	0.125	0.03	Wildtype	MAT1-2	B
C275	0.125	0.125	0.06	Wildtype	MAT1-2	B
C341	>16	0.5	0.25	TR_{34}/L98H	MAT1-1	A
C342	>16	0.5	0.25	TR_{34}/L98H	MAT1-2	A
C343	0.06	0.06	0.03	Wildtype	MAT1-2	B
C344	0.125	0.06	0.03	Wildtype	MAT1-1	B
C345	0.25	0.06	0.03	Wildtype	MAT1-2	B
C356	0.25	0.06	0.03	Wildtype	MAT1-2	B
C354	4	2	0.5	TR_{34}/L98H	MAT1-2	A
C355	2	2	0.5	TR_{34}/L98H	MAT1-1	A
C356	0.5	2	0.5	TR_{34}/L98H	MAT1-2	A
C357	ND	ND	ND	TR_{34}/L98H	MAT1-1	A
C358	ND	ND	ND	TR_{46}/Y121F/T289A	MAT1-2	A
C359	32	2	0.5	TR_{34}/L98H	MAT1-2	A
C360	ND	ND	ND	TR_{46}/Y121F/T289A	MAT1-2	A
C361	ND	ND	ND	Wildtype	MAT1-2	A
C362	0.75	0.125	0.25	Wildtype	MAT1-2	A
C363	0.03	0.12	0.25	Wildtype	MAT1-2	A
C364	4	0.5	ND	TR_{34}/L98H	MAT1-1	A
C365	16	2	ND	TR_{34}	MAT1-1	A
C366	4	0.25	ND	TR_{34}/L98H	MAT1-1	A
C367	1	0.5	ND	Wildtype	MAT1-2	A
C368	4	0.5	ND	TR_{34}/L98H	MAT1-2	A
C369	1	0.5	ND	TR_{34}/L98H	MAT1-1	A

Table S3

A. fumigatus isolates presented in this study with raised MICs to antifungal azole drugs, yet with no known resistance polymorphism within *cyp51A*. ND = not done.

Isolate	ITC MIC (mg/litre)	VOR MIC (mg/litre)	POS MIC (mg/litre)
C117	1	2	ND

C118	8	1	ND
C127	>16	0.25	0.125
C128	>16	2	1
C129	8	2	0.5
C136	>16	2	2
C137	>16	2	2
C139	>16	2	1
C148	2	0.125	0.03
C162	2	2	1
C164	2	1	0.125
C165	2	0.5	0.125
C168	2	0.25	0.03

Table S4

Relative frequencies of *cyp51A* genotypes and mating-type idiomorphs for clinical and environmental *A. fumigatus* isolates in this study

Cyp51A genotype	Number of clinical isolates (% of clinical isolates)	Number of environmental isolates (% of environmental isolates)
TR_{34}/L98H	44 (28.8%)	41 (63.1%)
TR_{34}	0	1 (1.5%)
TR_{34}/L98H/T289A/I364V/G448S	4 (2.6%)	0
TR_{46}/Y121F/T289A	0	7 (10.8%)
P216L	2 (1.3%)	0
G54W	9 (5.9%)	0
G54E	1 (0.6%)	0
G54R	2 (1.3%)	1 (1.5%)
Wildtype	91 (59.5%)	15 (23.1%)
MAT1-1	64 (41.8%)	15 (23.1%)
MAT1-2	89 (56.2%)	50 (76.9%)

Table S5

Relative frequencies of metadata associated with Clades A and B within this dataset

	Clade A (total 123 isolates)	Clade B (total 95 isolates)
Number of clinical isolates	71 (58%)	82 (86%)
Number of environmental isolates	52 (42%)	13 (14%)
MAT1-1	48 (39%)	31 (33%)
MAT1-2	75 (61%)	64 (66%)
Cyp51A wildtype	24 (20%)	82 (86%)
Cyp51A AMR polymorphisms	99 (80%)	13 (14%)

Table S6

Metadata for non-UK isolates added to phylogenetic analyses to confirm assignment of isolates into two clades, A and B

Isolate	Source	Location	*Cyp51A* polymorphisms	Date of isolation	Reference	Project accession
DRX13572	Clinical	Japan	Wildtype	2009	Takahashi-Nakaguchi *et al.* 2015	DRA001281
DRX13573	Clinical	Japan	Wildtype	2009	Takahashi-Nakaguchi *et al.* 2015	DRA001281
DRX13574	Clinical	Japan	Wildtype	2007	Takahashi-Nakaguchi *et al.* 2015	DRA001281
DRX13575	Clinical	Japan	Wildtype	2009	Takahashi-Nakaguchi *et al.* 2015	DRA001281
DRX13576	Clinical	Japan	Wildtype	2011	Takahashi-Nakaguchi *et al.* 2015	DRA001281
DRX13677	Clinical	Japan	Wildtype	2009	Takahashi-Nakaguchi *et al.* 2015	DRA001281
DRX13578	Clinical	Japan	Wildtype	2012	Takahashi-Nakaguchi *et al.* 2015	DRA001281
DRX13579	Clinical	Japan	Wildtype	2012	Takahashi-Nakaguchi *et al.* 2015	DRA001281
DRX15829	Clinical	Japan	Wildtype	2009	Takahashi-Nakaguchi *et al.* 2015	DRA001281
DRX15830	Clinical	Japan	Wildtype	2009	Takahashi-Nakaguchi *et al.* 2015	DRA001281
DRX15831	Clinical	Japan	Wildtype	2009	Takahashi-Nakaguchi *et al.* 2015	DRA001281
DRX15832	Clinical	Japan	Wildtype	2010	Takahashi-Nakaguchi *et al.* 2015	DRA001281
DRX15833	Clinical	Japan	Wildtype	2010	Takahashi-Nakaguchi *et al.* 2015	DRA001281
DRX15834	Clinical	Japan	Wildtype	2012	Takahashi-Nakaguchi *et al.* 2015	DRA001281
DRX15835	Clinical	Japan	Wildtype	2012	Takahashi-Nakaguchi *et al.* 2015	DRA001281

DRX15836	Clinical	Japan	Wildtype	2013	Takahashi-Nakaguchi *et al.* 2015	DRA001281
DRX15837	Clinical	Japan	Wildtype	2014	Takahashi-Nakaguchi *et al.* 2015	DRA001281
IFM59355-1	Clinical	Japan	Wildtype	2009	Hagiwara *et al.* 2014	PRJDB3064
IFM59355-2	Clinical	Japan	Wildtype	2009	Hagiwara *et al.* 2014	PRJDB3064
IFM59356-1	Clinical	Japan	Wildtype	2009	Hagiwara *et al.* 2014	PRJDB3064
IFM59356-2	Clinical	Japan	Wildtype	2009	Hagiwara *et al.* 2014	PRJDB3064
IFM59356-3	Clinical	Japan	Wildtype	2009	Hagiwara *et al.* 2014	PRJDB3064
IFM59361-1	Clinical	Japan	Wildtype	2009	Hagiwara *et al.* 2014	PRJDB3064
IFM59361-2	Clinical	Japan	Wildtype	2009	Hagiwara *et al.* 2014	PRJDB3064
IFM60237	Clinical	Japan	P216L	2011	Hagiwara *et al.* 2014	PRJDB3064
08-12-12-13	Clinical	The Netherlands	TR_{34}/L98H	2003	Abdolrasouli *et al.* 2015	PRJEB8623
08-36-03-25	Clinical	The Netherlands	TR_{34}/L98H	2005	Abdolrasouli *et al.* 2015	PRJEB8623
08-31-08-91	Clinical	The Netherlands	TR_{34}/L98H	2004	Abdolrasouli *et al.* 2015	PRJEB8623
10-01-02-27	Clinical	The Netherlands	TR_{34}/L98H	2010	Abdolrasouli *et al.* 2015	PRJEB8623
Afu_942_09	Clinical	India	TR_{34}/L98H	2009	Abdolrasouli *et al.* 2015	PRJEB8623
Afu_1942_09	Clinical	India	TR_{34}/L98H	2009	Abdolrasouli *et al.* 2015	PRJEB8623
Afu_343_P_11	Clinical	India	TR_{34}/L98H	2011	Abdolrasouli *et al.* 2015	PRJEB8623
Afu_591_12	Clinical	India	TR_{34}/L98H	2012	Abdolrasouli *et al.* 2015	PRJEB8623
08-19-02-61	Environmental	The Netherlands	TR_{34}/L98H	2008	Abdolrasouli *et al.* 2015	PRJEB8623

08-19-02-30	Environmental	The Netherlands	Wildtype	2008	Abdolrasouli *et al.* 2015	PRJEB8623
08-19-02-46	Environmental	The Netherlands	TR_{34}/L98H	2008	Abdolrasouli *et al.* 2015	PRJEB8623
08-19-02-10	Environmental	The Netherlands	TR_{34}/L98H	2008	Abdolrasouli *et al.* 2015	PRJEB8623
Afu_124_E11	Environmental	India	TR_{34}/L98H	2011	Abdolrasouli *et al.* 2015	PRJEB8623
Afu_166_E11	Environmental	India	TR_{34}/L98H	2011	Abdolrasouli *et al.* 2015	PRJEB8623
Afu_257_E11	Environmental	India	TR_{34}/L98H	2011	Abdolrasouli *et al.* 2015	PRJEB8623
Afu_218_E11	Environmental	India	TR_{34}/L98H	2011	Abdolrasouli *et al.* 2015	PRJEB8623

Table S7

Average value of Tajima's *D* statistic per chromosome for isolates within Clades A and B, and average and F_{ST} values per chromosome. Range of values are shown in brackets.

Chromosome	Clade A	Clade B	F_{ST}
I	0.4047 (-1.9766-3.9560)	-0.5535 (-2.6785-2.6761)	0.1022 (0-0.9440)
II	0.5241 (-2.4022-5.5133)	-0.3028 (-2.0764-4.8010)	0.1156 (0.0069-0.3539)
III	0.3267 (-2.1441-4.3288)	-0.3422 (-2.5142-3.2920)	0.1273 (0.0092-0.4244)
IV	0.2774 (-2.1356-3.9701)	-0.4883 (-2.4272-3.7844)	0.1622 (0.0115-0.5633)
V	0.3775 (-2.26076-4.3397)	-0.3317 (-2.2117-3.4575)	0.1616 (0.0168-0.5171)
VI	0.7591 (-2.0187-4.1077)	-0.5145 (-2.5173-3.6985)	0.1055 (0.0131-0.4314)
VII	0.7888 (-2.0023-4.1684)	-0.2754 (-2.6883-3.8941)	0.1530 (0.0208-0.5591)

VIII	0.6067 (-2.0557-4.4425)	-0.0396 (-2.2204-3.1474)	0.1530 (0.0202-0.3710)
Mitochondria (MT)	0.5311 (-1.0274-2.6496)	-0.7255 (-0.9931- -0.2730)	0.1520 (0.0682-0.2765)

Table S8

Genes found within regions of high F_{ST} in Chromosome 1 when performing fixation index analysis between Clades A and B in non-overlapping windows of 10 kb

Gene ID	Genomic Location	Product Description
Afu1g15000	4,029,090 – 4,031,693	Putative isopropylmalate synthase
Afu1g15010	4,031,939 – 4,038,777	Ortholog of *A. flavus* putative AMP binding domain protein AFLA_084630
Afu1g15020	4,039,173 – 4,040,722	40S ribosomal protein S5
Afu1g16860	4,598,911 – 4,600,377	Ortholog of *A. flavus* conserved hypothetical protein ALFA_082490
Afu1g16870	4,600,954 – 4,602,774	Ortholog of *A. flavus* conserved hypothetical protein AFLA_016260
Afu1g16880	4,603,153 – 4,608,184	Ortholog of *A. flavus* putative ABC multidrug transporter AFLA_082400
Afu1g16890	4,608,535 – 4,609,830	Ortholog of *A. flavus* putative transesterase LovD AFLA_063440
Afu1g16900	4,615,099 – 4,617,349	Ortholog of *A. fumigatus* A1163 AFUB_016290 (protein of unknown function)
Afu1g16910	4,617,552 – 4,619,374	Has domain with predicted role in transmembrane transport

Table S9

Pairs or groups of *A. fumigatus* isolates from both environmental and clinical sources with high genetic relatedness.

Pair/ Group	Isolates included (Clade)	Mating idiomorph	Source	*cyp51A* allele	Bootstrap support	Average SNPs separating isolates	One tailed t-test *p*-value
1	C21 (A_A)	MAT1-2<br>MAT1-2	Environmental<br>Clinical	TR_{34}/L98H<br>TR_{34}/L98H	100%	227	<3.0473^{e-252}

	C112<br>(A_A)						
2	C133<br>(A_A)<br>C23 (A_A)	MAT1-2<br>MAT1-2	Clinical<br>Environmental	TR$_{34}$/L98H<br>TR$_{34}$/L98H	65%	270	<3.0473^{e-252}
3	C354 (A)<br>C355 (A)<br>C357 (A)	MAT1-2<br>MAT1-1<br>MAT1-1	Clinical<br>Clinical<br>Environmental	TR$_{34}$/L98H<br>TR$_{34}$/L98H<br>TR$_{34}$/L98H	100%	237	<3.0473^{e-252}
4	C191 (B)<br>C275 (B)	MAT1-2<br>MAT1-2	Clinical<br>Environmental	Wildtype<br>Wildtype	100%	581	<3.0473^{e-252}
5	C96 (B)<br>C42 (B)<br>C43 (B)<br>C44 (B)<br>C48 (B)	MAT1-2<br>MAT1-2<br>MAT1-2<br>MAT1-2<br>MAT1-2	Environmental<br>Clinical<br>Clinical<br>Clinical<br>Clinical	Wildtype<br>Wildtype<br>Wildtype<br>Wildtype<br>Wildtype	79%	217	3.0473^{e-252}
6	C7 (A)<br>C25 (A)<br>C82 (A)	MAT1-2<br>MAT1-2<br>MAT1-2	Environmental<br>Environmental<br>Clinical	TR$_{34}$/L98H<br>TR$_{34}$/L98H<br>TR$_{34}$/L98H	100%	252	1.7857^{e-112}

Table S10

Essential Af293 genes are found within the pangenome but vary amongst the population

Pangenome gene ID	Af293 gene ID	Number of genomes
centroid_9134	AFUA_2G09060	156
centroid_9784	AFUA_4G11280	173
centroid_4480	AFUA_3G12290	215
centroid_6938	AFUA_6G04740	215
centroid_2065	AFUA_2G07570	216
centroid_4310	AFUA_2G06150	216
centroid_8665	AFUA_6G06810	217
centroid_904	AFUA_1G13100	217
centroid_1959	AFUA_2G12370	217
centroid_7884	AFUA_3G08080	217
centroid_3136	AFUA_1G14740	217
centroid_8331	AFUA_2G03310	217

Supplementary Figure legends

Extended Data Figure 1: Microreact project screenshot of the dataset https://microreact.org/project/viUDBzrCmTNKmY9Fu6Zhxi

Extended Data Figure 2: Phylogenetic analysis of all 218 *A. fumigatus* isolates with bootstrap support over 1000 replicates performed on WGS SNP data to generate maximum-likelihood phylogeny. Branch lengths represent average number of SNPs.

Extended Data Figure 3: Phylogenetic analysis of all 218 *A. fumigatus* isolates plus an additional 41 publicly available WGS of non-UK origin confirms the clade assignment into Clades A and B was not an artifact of these data but also seen globally. These additional data comprised of 33 clinical and 8 environmental isolates. Maximum-likelihood phylogeny generated with bootstrap support over 1000 replicates on WGS SNP data.

Extended Data Figure 4: Microreact project screenshot of the dataset with DAPC clusters showing lack of geographic and temporal clustering.

Extended Data Figure 5: Scatterplots of sliding 10-kb non-overlapping window estimates of F_{ST} for each chromosome between isolates within Clusters 1 and 2, Clusters 1 and 3, and Clusters 2 and 3 (top to bottom, left panel). Scatterplots of Tajima's D estimates for each chromosome for all isolates within Clusters 1, 2 and 3 (right panel). The position of *cyp51A* is highlighted in red.

Supplementary Data

Supplementary Data 1 (Excel format): Significant SNPs for itraconazole resistance using the TreeWAS subsequent association test, in order of ascending p-value for all *A. fumigatus* isolates in this study with known itraconazole MICs. Chromosome, SNP position and gene loci listed along with gene description and type of SNP (synonymous or non-synonymous).

Supplementary Data 2 (Excel format): Significant SNPs for itraconazole resistance using the TreeWAS terminal association test, in order of ascending p-value for all *A. fumigatus* isolates in this study with known itraconazole MICs. Chromosome, SNP position and gene loci listed along with gene description and type of SNP (synonymous or non-synonymous).

Supplementary Data 3 (Excel format): Significant SNPs for itraconazole resistance using the TreeWAS simultaneous association test, in order of ascending p-value for all *A. fumigatus* isolates in this study with known itraconazole MICs. Chromosome, SNP position and gene loci listed along with gene description and type of SNP (synonymous or non-synonymous).

Supplementary Data 4 (Excel format): Summary of 184 genes found in region of high F_{ST} in chromosome 1 when comparing clades A and B. Associated *p*-values from TreeWAS for genes containing SNPs that are statistically significant for itraconazole resistance are also included.

Supplementary Data 5, 6 and 7 (Excel format): filtered Scoary results of all genes associated with Clade A vs. B (5), azole drug resistance (6) and mutation vs. wildtype (7) from pangenome analysis.
