# Supplementary material for: Population genomics confirms acquisition of drug-resistant Aspergillus fumigatus infection by humans from the environment
Source: Nat Microbiol. 2022 Apr 25;7(5):663–74. doi: 10.1038/s41564-022-01091-2 (PMC9064804; doi:10.1038/s41564-022-01091-2)
Supplement: Supplementary file 2 — Reporting Summary [file 41564_2022_1091_MOESM2_ESM.pdf]

nature research

Corresponding author(s): Johanna Rhodes

Last updated by author(s): 17th Feb 2022

Reporting Summary

Nature Research wishes to improve the reproducibility of the work that we publish. This form provides structure for consistency and transparency in reporting. For further information on Nature Research policies, see Authors & Referees and the Editorial Policy Checklist.

Statistics

For all statistical analyses, confirm that the following items are present in the figure legend, table legend, main text, or Methods section.

n/a	Confirmed	
☐	☒	The exact sample size (n) for each experimental group/condition, given as a discrete number and unit of measurement
☐	☒	A statement on whether measurements were taken from distinct samples or whether the same sample was measured repeatedly
☐	☒	The statistical test(s) used AND whether they are one- or two-sided<br>*Only common tests should be described solely by name; describe more complex techniques in the Methods section.*
☐	☒	A description of all covariates tested
☐	☒	A description of any assumptions or corrections, such as tests of normality and adjustment for multiple comparisons
☐	☒	A full description of the statistical parameters including central tendency (e.g. means) or other basic estimates (e.g. regression coefficient) AND variation (e.g. standard deviation) or associated estimates of uncertainty (e.g. confidence intervals)
☐	☒	For null hypothesis testing, the test statistic (e.g. F, t, r) with confidence intervals, effect sizes, degrees of freedom and P value noted<br>*Give P values as exact values whenever suitable.*
☐	☒	For Bayesian analysis, information on the choice of priors and Markov chain Monte Carlo settings
☒	☐	For hierarchical and complex designs, identification of the appropriate level for tests and full reporting of outcomes
☒	☐	Estimates of effect sizes (e.g. Cohen's d, Pearson's r), indicating how they were calculated

Our web collection on statistics for biologists contains articles on many of the points above.

Software and code

Policy information about availability of computer code

Data collection	The whole genome sequence data were generated via the Illumina HiSeq sequencing platform
Data analysis	The WGS data were aligned to reference sequence using a custom pipeline as described in Rhodes et al 2015 (https://journals.asm.org/doi/abs/10.1128/mBio.00536-15)

For manuscripts utilizing custom algorithms or software that are central to the research but not yet described in published literature, software must be made available to editors/reviewers. We strongly encourage code deposition in a community repository (e.g. GitHub). See the Nature Research guidelines for submitting code & software for further information.

Data

Policy information about availability of data

All manuscripts must include a data availability statement. This statement should provide the following information, where applicable:

- Accession codes, unique identifiers, or web links for publicly available datasets
- A list of figures that have associated raw data
- A description of any restrictions on data availability

Source data are provided in the paper, its supplementary files, and a Source Data file for all Figures. The WGS data are deposited in the EBI-ENA under project accession PRJEB27135. Custom scripts for pangenome analysis can be found at github.com/harrychown/asp_pan

Field-specific reporting

Please select the one below that is the best fit for your research. If you are not sure, read the appropriate sections before making your selection.

☒ Life sciences ☐ Behavioural & social sciences ☐ Ecological, evolutionary & environmental sciences

For a reference copy of the document with all sections, see nature.com/documents/nr-reporting-summary-flat.pdf

Life sciences study design

All studies must disclose on these points even when the disclosure is negative.

Sample size	Many isolates from both clinical and environmental sources were specifically selected for whole genome sequencing because they displayed phenotypic azole resistance (raised minimum inhibitory concentrations (MICs) to at least one triazole drug using EUCAST or CLSI) and do not constitute a randomised sample.
Data exclusions	Data from contaminated samples, e.g. contaminated seedlings and plates, were excluded.
Replication	Where multiple sequencing runs were used, a replicate of a single isolate was included to ensure no variation due to sequencing machine
Randomization	Samples were chosen randomly from each genotype per treatment per time point.
Blinding	Investigators were blinded with regards personal information and clinical data.

Reporting for specific materials, systems and methods

We require information from authors about some types of materials, experimental systems and methods used in many studies. Here, indicate whether each material, system or method listed is relevant to your study. If you are not sure if a list item applies to your research, read the appropriate section before selecting a response.

Materials & experimental systems

n/a	Involved in the study	
☒	☐	Antibodies
☒	☐	Eukaryotic cell lines
☒	☐	Palaeontology
☒	☐	Animals and other organisms
☒	☐	Human research participants
☒	☐	Clinical data

Methods

n/a	Involved in the study	
☒	☐	ChIP-seq
☒	☐	Flow cytometry
☒	☐	MRI-based neuroimaging

Antibodies

Antibodies used	N/A
Validation	
